# Gender disparity in admissions into tertiary institutions: Empirical evidence from Nigerian data (2010–2015)

**DOI:** 10.1016/j.dib.2019.01.031

**Published:** 2019-01-18

**Authors:** Olumuyiwa A. Oludayo, Segun I. Popoola, Comfort O. Akanbi, Aderemi A. Atayero

**Affiliations:** a Department of Business Management, Covenant University, Ota, Nigeria; b IoT-Enabled Smart and Connected Communities (SmartCU) Research Cluster, Department of Electrical and Information Engineering, Covenant University, Ota, Nigeria; c Department of Economics, Bowen University, Iwo, Nigeria

## Abstract

Gender equality in access to higher education is an important factor in building a sustainable world. Although a good number of countries across the globe have achieved parity in primary education between boys and girls, the target is yet to be widely attained at tertiary level of education. In this data article, empirical data on yearly admissions into accredited tertiary institutions in Nigeria are extensively explored to reveal the existence of gender gaps in the national admission process. Details on the number of candidates admitted into all accredited universities, polytechnics, and colleges of education between 2010 and 2015 were obtained directly from the Joint Admissions and Matriculation Board (JAMB). Gender distributions of admitted candidates are analyzed across the thirty-six (36) states of the federation, the Federal Capital Territory (FCT), and the international students’ category. Gender disparity in admissions into Nigerian tertiary institutions are explored using relevant descriptive statistics, box plots, bar charts, line graphs, and pie charts. In addition, Analysis of Variance (ANOVA) is carried out on the historical data to find out if there are significant differences in the arithmetic means of females and males admitted over the six-year period. Furthermore, multiple comparison post-hoc test results are presented in tables to understand the extent of variations (if any) in gender distribution over the years. The robust data exploration reported in this data article will help national regulatory bodies and relevant stakeholders in policy formulation and decision making towards ensuring equal access to higher education in Nigeria.

**Specifications table**

**Table:** 

Subject area	*Education*
More specific subject area	*Gender gap analysis in tertiary education*
Type of data	*Tables, charts, and spreadsheet file tables*
How data were acquired	*Details on the number of candidates admitted into all accredited universities, polytechnics, and colleges of education between 2010 and 2015 were obtained directly from JAMB.*
Data format	*Secondary, analyzed*
Experimental factors	*The admission data of universities, polytechnics, and colleges of education that are yet to be accredited by respective national regulatory bodies were excluded.*
Experimental features	*Gender disparity in admissions into Nigerian tertiary institutions are explored using relevant descriptive statistics, ANOVA, and multiple comparison post-hoc test. The results are presented in form of box plots, bar charts, line graphs, and pie charts.*
Data source location	*Nigeria, West Africa (Latitude 9.0820°N, Longitude 8.6753°E)*
Data accessibility	https://data.mendeley.com/datasets/9w7c3xtv9x/1
	http://dx.doi.org/10.17632/9w7c3xtv9x.1
Related research article	*E. Lahelma, "Troubling discourses on gender and education," Educational Research, vol. 56, pp. 171–183, 2014*[1]

**Value of the data**

• Thorough evaluation, correct interpretation, and contextual discussion of the data analyses provided in this data article may speed up the achievement of Goals 4 and 5 of the global Sustainable Development Goals (SDGs) in Nigeria.
• Various research efforts aimed at achieving SDGs 1, 3, 7, 10, and 11, will be intensified if access is granted to more females into Higher Education Institutions, particularly into Science, Technology, Engineering, and Mathematics (STEM) programs.
• The data exploration provided in this data article will call the attention of national regulatory bodies, executives, administrators, and relevant stakeholders to the need for strategic advocacy and promotion of equal access to higher education among all women and men in Nigeria.
• Robust data exploration presented in this data article will help the United Nations (UN) to objectively assess the level of gender disparity in access to higher education in Nigeria.
• The contribution of this data article will further widen the coverage of evidence-based and reproducible research on gender disparity in higher education. In addition, the utility of the data may open doors for new research collaborations on the trends and patterns of enrollment by gender in higher institutions in sub-Saharan Africa.

## 1 Data

Elimination of gender disparity at all levels of education is one of the major global goals in the pursuit of sustainable development across the globe [1], [2]. Although, a good number of countries across the globe have achieved parity in primary education between boys and girls, the target is yet to be widely attained at tertiary level of education [3], [4]. In this data article, empirical data on yearly admissions into accredited tertiary institutions in Nigeria are extensively explored to reveal the existence of gender gaps in the national admission process. Thorough evaluation, correct interpretation, and contextual discussion of the data analyses provided in this data article may speed up the achievement of Goals 4 and 5 of the global Sustainable Development Goals (SDGs) in Nigeria. The data exploration provided in this data article will call the attention of national regulatory bodies, executives, administrators, and relevant stakeholders to the need for strategic advocacy and promotion of equal access to higher education among all women and men in Nigeria. Robust data exploration presented in this data article will help the United Nations (UN) to objectively assess the level of gender disparity in access to higher education in Nigeria. The contribution of this data article will further widen the coverage of evidence-based and reproducible research on gender disparity in higher education. In addition, the utility of the data may open doors for new research collaborations on the trends and patterns of enrollment by gender in higher institutions in sub-Saharan Africa.

The basic features of the quantitative data on yearly admissions into all accredited Nigerian universities, polytechnics, and colleges of education between 2010 and 2015 were described using eleven (11) different statistical parameters namely: mean, median, mode, standard deviation, variance, kurtosis, Skewness, range, minimum value, maximum value, and the sum. The descriptive statistics of the number of candidates admitted are computed for two gender category (i.e. female and male) and the results obtained are presented in Table 1. The results of the descriptive statistics showed that there is gender disparity in admissions into tertiary institutions in Nigeria. In each of the six-year period, the number of males admitted into the various higher institutions of learning in Nigeria is consistently higher than that of their female counterparts.

**Table 1:** Descriptive statistics of candidates admitted into Nigerian tertiary institutions (2010–2015).

	**2010**		**2011**		**2012**		**2013**		**2014**		**2015**	
	*Female*	*Male*	*Female*	*Male*	*Female*	*Male*	*Female*	*Male*	*Female*	*Male*	*Female*	*Male*
**Mean**	3831.89	5475.92	993.97	1592.76	4536.95	6256.66	4468.68	6064.71	4160.11	5834.45	4687.08	6247.13
**Median**	3587.50	5376.00	811.50	1516.50	4062.00	6349.50	3594.00	5827.50	3230.50	5425.00	4209.00	5665.00
**Mode**	3.00	74.00	1420.00	24.00	45.00	0.00	13.00	23.00	25.00	23.00	20.00	20.00
**Standard Deviation**	3450.06	3847.82	871.31	1108.90	3614.41	4483.82	3647.43	3375.72	3351.50	3325.07	3419.58	3307.20
**Variance**	1.19 × 10^7^	1.48 × 10^7^	7.59 × 10^5^	1.23 × 10^6^	1.31 × 10^7^	2.01 × 10^7^	1.33 × 10^7^	1.14 × 10^7^	1.12 × 10^7^	1.11 × 10^7^	1.17 × 10^7^	1.09 × 10^7^
**Kurtosis**	2.63	1.83	2.70	1.99	2.34	2.97	2.80	1.93	2.14	1.86	2.31	2.08
**Skewness**	0.75	0.35	0.73	0.35	0.57	0.53	0.75	0.13	0.57	0.14	0.51	0.13
**Range**	12,598	12,922	3223	3946	13,069	18,996	14,311	12,650	11,253	12,375	13,073	12,602
**Minimum**	3	74	21	24	45	0	13	23	25	23	20	20
**Maximum**	12,601	12,996	3244	3970	13,114	18,996	14,324	12,673	11,278	12,398	13,093	12,622
**Sum**	145,612	208,085	37,771	60525	172,404	237,753	169,810	230,459	158,084	221,709	178,109	237,391

**Fig. 1:**
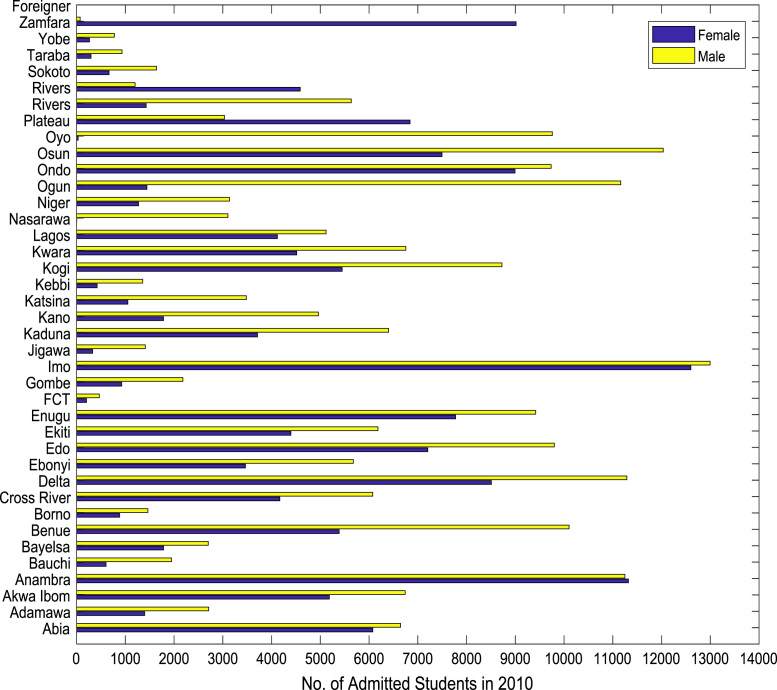
Gender distribution of higher education enrollment in 2010.

**Fig. 2:**
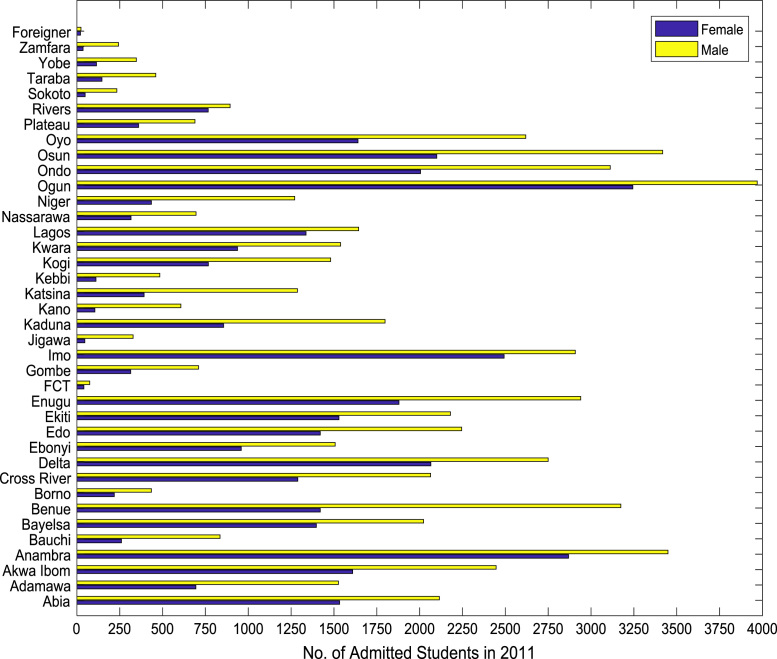
Gender distribution of higher education enrollment in 2011.

**Fig. 3:**
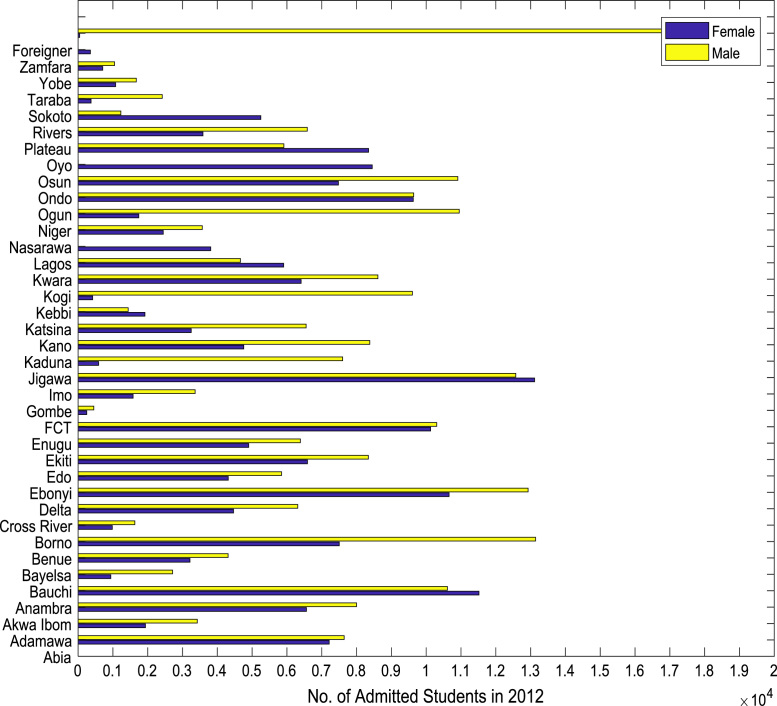
Gender distribution of higher education enrollment in 2012.

**Fig. 4:**
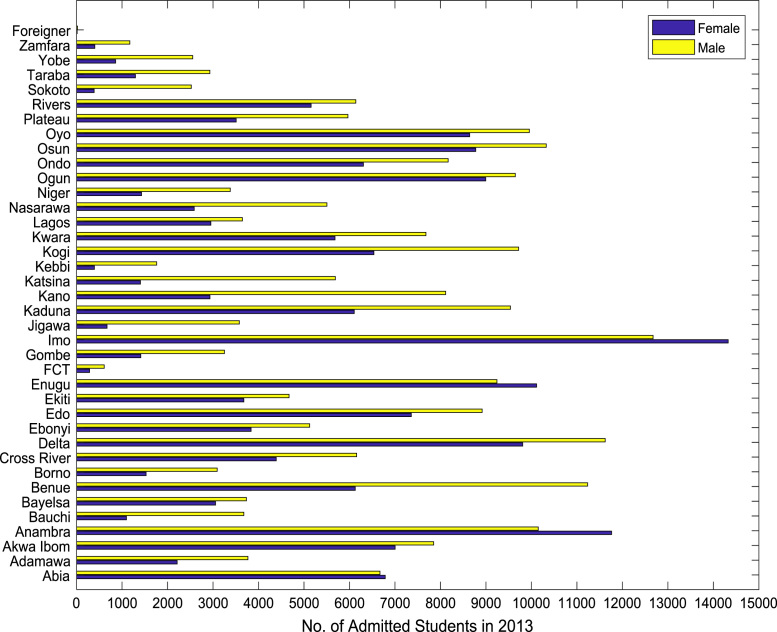
Gender distribution of higher education enrollment in 2013.

**Fig. 5:**
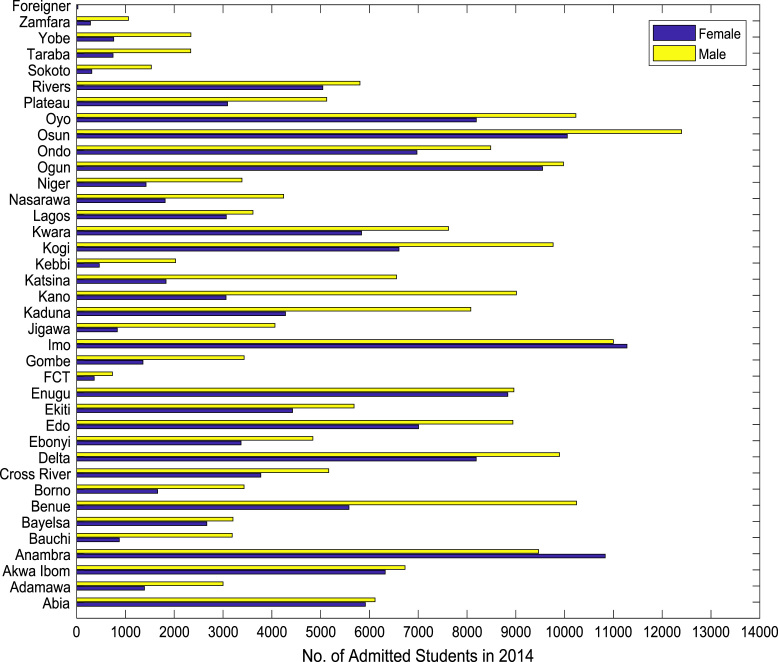
Gender distribution of higher education enrollment in 2014.

**Fig. 6:**
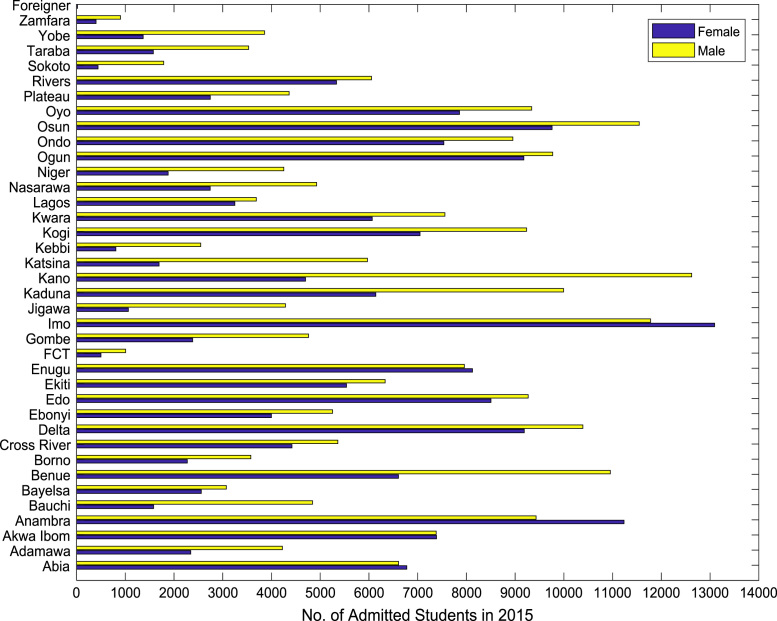
Gender distribution of higher education enrollment in 2015.

**Fig. 7:**
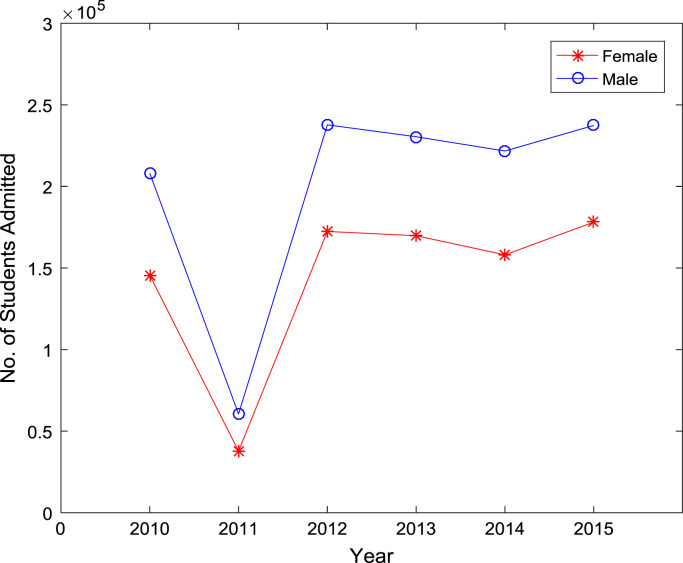
Time series plot of higher education enrollment by gender.

Also, the gender distributions of admitted candidates were analyzed across the thirty-six (36) states of the federation, the Federal Capital Territory (FCT), and the international students’ category. The distributions of enrollment in higher education for the period of six years (2010–2015) are shown in Fig. 1, Fig. 2, Fig. 3, Fig. 4, Fig. 5, Fig. 6 respectively. In order to gain better understanding of the trends and patterns of the gender inequality, the numbers of females and males admitted were plotted against the years of admission as shown in Fig. 7.

**Fig. 8:**
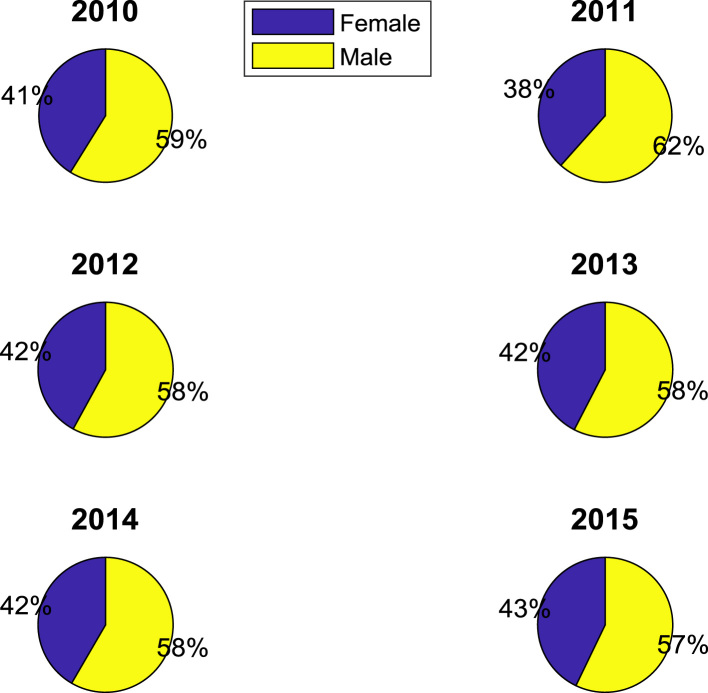
Proportions of females and males in admissions into Nigerian tertiary institutions.

**Fig. 9:**
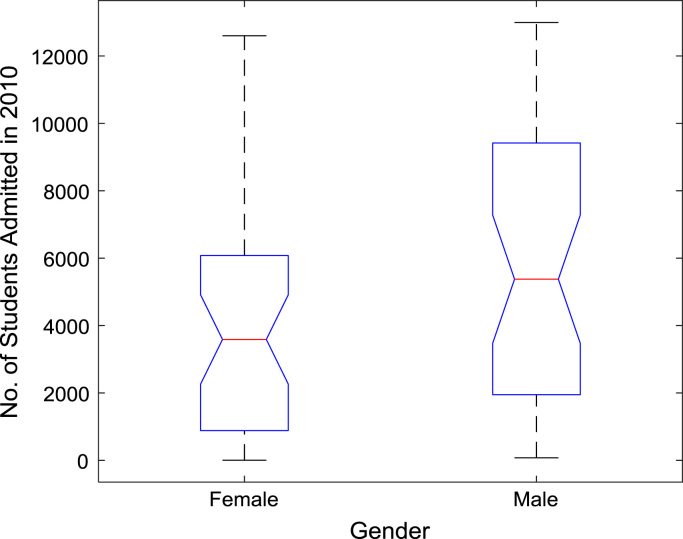
Boxplot representation of enrollment in 2010.

**Fig. 10:**
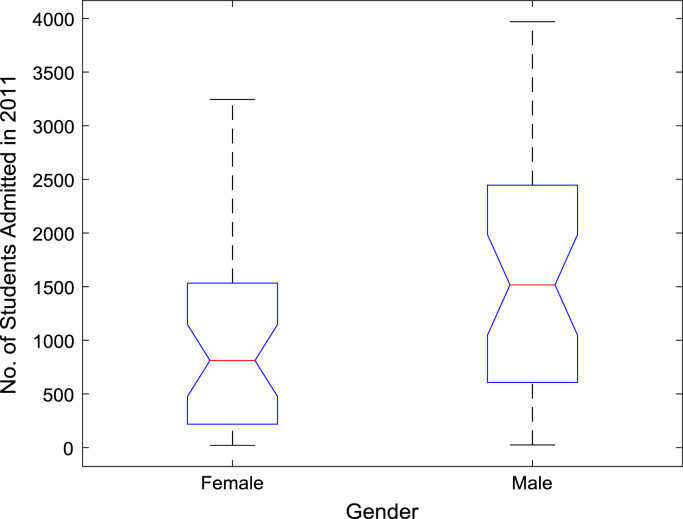
Boxplot representation of enrollment in 2011.

**Fig. 11:**
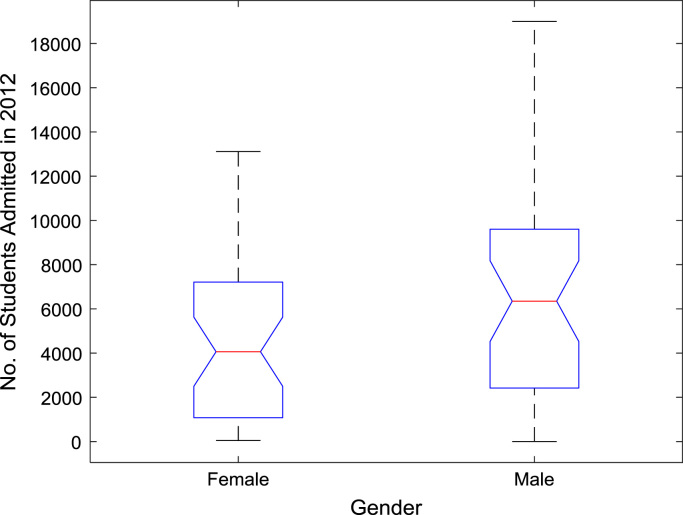
Boxplot representation of enrollment in 2012.

**Fig. 12:**
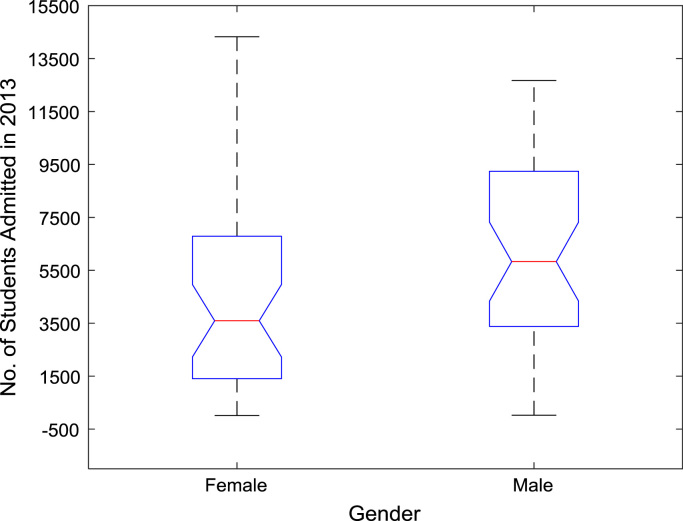
Boxplot representation of enrollment in 2013.

**Fig. 13:**
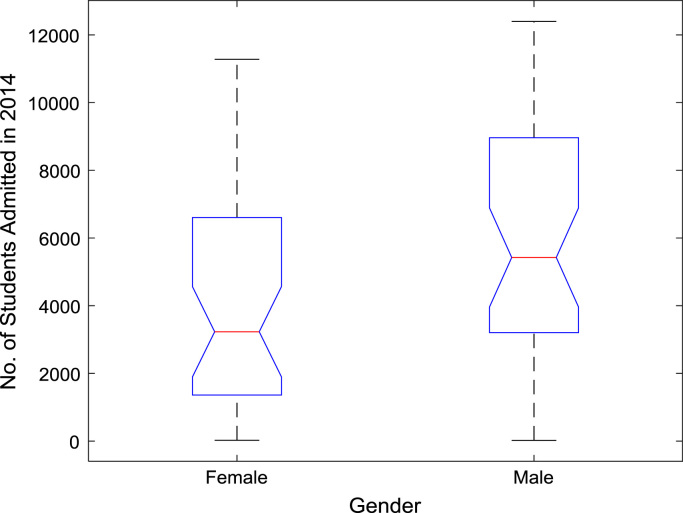
Boxplot representation of enrollment in 2014.

**Fig. 14:**
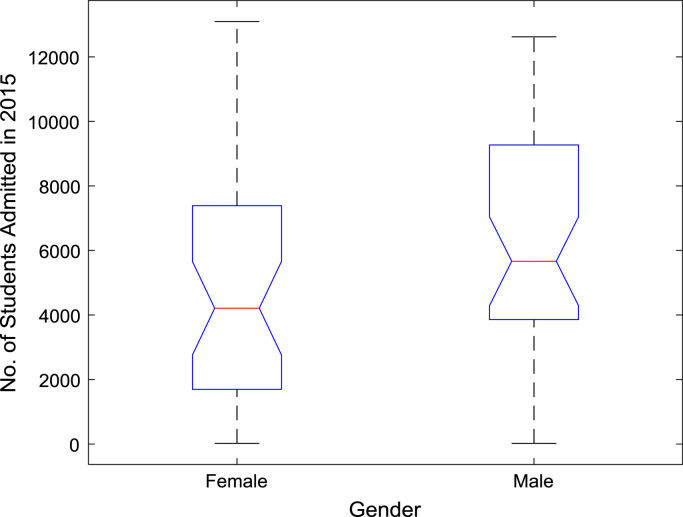
Boxplot representation of enrollment in 2015.

The proportions of females and males in admissions into Nigerian tertiary institutions are depicted using pie charts shown in Fig. 8. The male gender continuously dominated the admissions into higher education over the six-year period. ANOVA test was carried out on the historical data to find out if there are significant differences in the arithmetic means of females and males admitted over the six-year period. The source of variation, sum of squares, degree of freedom, mean squares, F-statistic, and the p-value are presented for each of the six-year period in Table 2, Table 3, Table 4, Table 5, Table 6, Table 7 respectively. Additional information on the variations between the arithmetic means over the years may be obtained from the boxplot representations shown in Fig. 9, Fig. 10, Fig. 11, Fig. 12, Fig. 13, Fig. 14. Furthermore, multiple comparison post-hoc test results are presented in Table 8 to understand the extent of variations (if any) in gender distribution over the years. Here, information about the lower limits for 95% confidence intervals, mean difference, upper limits for 95% confidence intervals, and p-value are provided.

**Table 2:** Gender gap ANOVA results of enrollment in 2010.

**Source of variation**	**Sum of squares**	**Degree of freedom**	**Mean squares**	**F statistic**		**Prob>F**
**Columns**	5.14 × 10^7^	1	5.14 × 10^7^		3.85	0.0536
**Error**	9.88 × 10^8^	74	1.34 × 10^7^			
**Total**	1.04 × 10^9^	75

**Table 3:** Gender gap ANOVA results of enrollment in 2011.

**Source of variation**	**Sum of squares**	**Degree of freedom**	**Mean squares**	**F statistic**	**Prob>F**
**Columns**	6.81 × 10^6^	1	6.81 × 10^6^	6.85	0.0107
**Error**	7.36 × 10^7^	74	9.94 × 10^5^		
**Total**	8.04 × 10^7^	75

**Table 4:** Gender gap ANOVA results of enrollment in 2012.

**Source of variation**	**Sum of squares**	**Degree of freedom**	**Mean squares**	**F statistic**		**Prob>F**
**Columns**	5.62 × 10^7^	1	5.62 × 10^7^		3.39	0.0697
**Error**	1.23 × 10^9^	74	1.66 × 10^7^			
**Total**	1.28 × 10^9^	75

**Table 5:** Gender gap ANOVA results of enrollment in 2013.

**Source of variation**	**Sum of squares**	**Degree of freedom**	**Mean squares**	**F statistic**		**Prob>F**
**Columns**	4.84 × 10^7^	1	4.84 × 10^7^		3.92	0.0515
**Error**	9.14 × 10^8^	74	1.23 × 10^7^			
**Total**	9.62 × 10^8^	75

**Table 6:** Gender gap ANOVA results of enrollment in 2014.

**Source of variation**	**Sum of squares**	**Degree of freedom**	**Mean squares**	**F statistic**		**Prob>F**
**Columns**	5.33 × 10^7^	1	5.33 × 10^7^		4.78	0.032
**Error**	8.25 × 10^8^	74	1.11 × 10^7^			
**Total**	8.78 × 10^8^	75

**Table 7:** Gender gap ANOVA results of enrollment in 2015.

**Source of variation**	**Sum of squares**	**Degree of freedom**	**Mean squares**	**F statistic**		**Prob>F**
**Columns**	4.62 × 10^7^	1	4.62 × 10^7^		4.09	0.0468
**Error**	8.37 × 10^8^	74	1.13 × 10^7^			
**Total**	8.84 × 10^8^	75

**Table 8:** Multiple comparison post-hoc test results.

**Year**	**Groups compared**		**Lower limits for 95% confidence intervals**	**Mean difference**	**Upper limits for 95% confidence intervals**	***p*****-value**
**2010**	Female	Male	−3314.510	−1644.026	26.457	0.054
**2011**	Female	Male	−1054.633	−598.789	−142.946	0.011
**2012**	Female	Male	−3581.283	−1719.711	141.862	0.070
**2013**	Female	Male	−3202.443	−1596.026	10.390	0.051
**2014**	Female	Male	−3200.352	−1674.342	−148.332	0.032
**2015**	Female	Male	−3314.510	−1644.026	26.457	0.054

## 2 Experimental design, materials, and methods

Details on the number of candidates admitted into all accredited universities, polytechnics, and colleges of education between 2010 and 2015 were obtained directly from the Joint Admissions and Matriculation Board (JAMB). Gender distributions of admitted candidates are analyzed across the thirty-six (36) states of the federation, the Federal Capital Territory (FCT), and the international students’ category. Gender disparity in admissions into Nigerian tertiary institutions are explored using relevant descriptive statistics, box plots, bar charts, line graphs, and pie charts.
