# Supplementary material for: Gender disparity in admissions into tertiary institutions: Empirical evidence from Nigerian data (2010–2015)
Source: Data Brief. 2019 Jan 18;22:920–33. doi: 10.1016/j.dib.2019.01.031 (PMC6362862; doi:10.1016/j.dib.2019.01.031)
Supplement: Supplementary file 3 — Supplementary material [file mmc3.pdf]

Covenant University
Canaanland, Km 10, Idiroko Road, Ota, Ogun State, Nigeria.
E-mail:cucrid@covenantuniversity.edu.ng
Website: www.covenantuniversity.edu.ng
Tel:+234-014542070

THE WORLD UNIVERSITY RANKINGS 2019 TOP 800

OFFICE OF THE DIRECTOR, COVENANT UNIVERSITY CENTRE FOR RESEARCH, INNOVATION AND DISCOVERY
Professor Nicholas A. I. Omoregbe

17th December, 2018.

The Editor-in-Chief,

Data in Brief,

3251 Riverport,

Maryland Heights,

MO 63043,

USA.

Dear Prof. Hao-Ran Wang,

LETTER OF INTRODUCTION: PROOF OF STUDY

The article titled ***Gender Disparity in Admissions into Tertiary Institutions: Empirical Evidence from Nigerian Data (2010-2015)*** submitted by ***Olumuyiwa A. Oludayo, Segun I. Popoola, Comfort O. Akanbi,*** and ***Aderemi A. Atayero*** for publication in your reputable journal has gone through the Ethical Committee of Covenant University. This letter serves as a proof that the study was performed in this university and its authenticity has been verified. The paper is hereby recommended for further processing.

Thank you.

For ______________________

Chair, Ethical Committee

____

Director, CUCRID
